# Proteomics Analysis of the DF-1 Chicken Fibroblasts Infected with Avian Reovirus Strain S1133

**DOI:** 10.1371/journal.pone.0092154

**Published:** 2014-03-25

**Authors:** Wen-Ting Chen, Yi-Le Wu, Ting Chen, Chao-Sheng Cheng, Hong-Lin Chan, Hsiu-Chuan Chou, Yi-Wen Chen, Hsien-Sheng Yin

**Affiliations:** 1 Institute of Bioinformatics and Structural Biology and College of Life Sciences, National Tsing Hua University, Hsinchu, Taiwan; 2 Department of Applied Science, National Hsinchu University of Education, Hsinchu, Taiwan; SRI International, United States of America

## Abstract

**Background:**

Avian reovirus (ARV) is a member of the *Orthoreovirus* genus in the Reoviridae family. It is the etiological agent of several diseases, among which viral arthritis and malabsorption syndrome are the most commercially important, causing considerable economic losses in the poultry industry. Although a small but increasing number of reports have characterized some aspects of ARV infection, global changes in protein expression in ARV-infected host cells have not been examined. The current study used a proteomics approach to obtain a comprehensive view of changes in protein levels in host cells upon infection by ARV.

**Methodology and Principal Findings:**

The proteomics profiles of DF-1 chicken fibroblast cells infected with ARV strain S1133 were analyzed by two-dimensional differential-image gel electrophoresis. The majority of protein expression changes (≥1.5 fold, *p*<0.05) occurred at 72 h post-infection. Matrix-assisted laser desorption ionization time-of-flight mass spectrometry identified 51 proteins with differential expression levels, including 25 that were upregulated during ARV infection and 26 that were downregulated. These proteins were divided into eight groups according to biological function: signal transduction, stress response, RNA processing, the ubiquitin-proteasome pathway, lipid metabolism, carbohydrate metabolism, energy metabolism, and cytoskeleton organization. They were further examined by immunoblotting to validate the observed alterations in protein expression.

**Conclusion/Significance:**

This is the first report of a time-course proteomic analysis of ARV-infected host cells. Notably, all identified proteins involved in signal transduction, RNA processing, and the ubiquitin-proteasome pathway were downregulated in infected cells, whereas proteins involved in DNA synthesis, apoptosis, and energy production pathways were upregulated. In addition, other differentially expressed proteins were linked with the cytoskeleton, metabolism, redox regulation, and stress response. These proteomics data provide valuable information about host cell responses to ARV infection and will facilitate further studies of the molecular mechanisms underlying ARV pathogenesis.

## Introduction

Avian reovirus (ARV), a member of the Reoviridae family, is a pathogenic agent in chicken (*Gallus gallus*) and is associated with several diseases such as viral arthritis and malabsorption syndrome, causing considerable economic losses in the poultry industry [1]. The primary method to control ARV infection in chickens is vaccination, but the vaccines do not protect animals from all antigenic subtypes [2]. The ARV genome contains ten segments of double-stranded RNA that encode at least eight structural proteins (λA, λB, λC, μA, μB, σA, σB, and σC) and four non-structural proteins (μNS, P10, P17, and σNS) [3]. The roles of some of these viral proteins in ARV replication and pathogenesis have been characterized. For instance, outer capsid protein μB is involved in viral entry [4]. Outer capsid protein σC is a cell attachment protein [5], and overexpression of σC in cultured cells can induce apoptosis [6]. Other studies have shown that the non-structural protein p10 is a multifunctional protein that not only causes syncytium formation in transfected cells [7], but also enhances membrane permeability [8]. More recent studies have reported a connection between ARV proteins and cellular signaling pathways. For example, non-structural protein p17 triggers autophagy, enhancing viral replication through the PKR/eIF2alpha signaling pathway [9], and capsid protein σC induces apoptosis via the DNA damage signaling pathway [10].

Although the functional significance of some individual ARV proteins has been examined, global changes in cell function during different stages of the ARV life cycle have not been explored. Recently, proteomic approaches incorporating two-dimensional differential-image gel electrophoresis (2D-DIGE) coupled with matrix-assisted laser desorption ionization time-of-flight mass spectrometry (MALDI-TOF MS) have been extensively utilized to monitor protein expression in response to viral infection [11]–[13]. These proteomic methods provide a comprehensive view of protein profiles of virus-infected host cells as well as insight into the molecular pathogenesis of viral infection. The proteins identified through this approach may also represent potential drug targets for antiviral therapy.

In this study, we report the first time-course analysis of ARV infection that couples a 2D-DIGE/MALDI-TOF-MS proteomic approach with confirmation of expression by western blotting. Further, we present the differential expression results in the context of biological processes that may play vital roles during ARV infection. This research provides new information and indicators to facilitate further studies of ARV pathogenesis and ARV–host interactions.

## Materials and Methods

### Chemicals and reagents

Most chemicals were purchased from Sigma-Aldrich (St. Louis, MO), except for reagents for 2D-DIGE, which were purchased from GE Healthcare (Uppsala, Sweden). Primary antibodies were purchased from Abcam (Cambridge, UK), except for anti-ARV, which was a kind gift from Dr. L. H. Lee at National Chung Hsing University, Taiwan. Secondary antibodies for immunofluorescence analysis, tetramethylrhodamine isothiocyanate−conjugated goat anti-rabbit IgG and fluorescein isothiocyanate−conjugated goat anti-mouse IgG, were purchased from Sigma-Aldrich. Secondary antibodies for western blotting, horseradish peroxidase−conjugated goat anti-mouse IgG and goat anti-rabbit IgG, were purchased from Jackson ImmunoResearch Laboratories (West Grove, PA).

### Cell culture and virus infection

The DF-1 cell line (ATCC CRL-12203) derived from chicken embryo fibroblasts [14] was obtained from Dr. C. H. Wang, Department of Veterinary Medicine, National Taiwan University, Taiwan. The cells were cultured in Dulbecco's modified Eagle medium (DMEM, Life Technologies, Grand Island, NY) supplemented with 10% fetal bovine serum, 100 U/ml penicillin, and 100 µg/ml streptomycin (Life Technologies) at 37°C in an atmosphere of 5% CO_2_ [15]. ARV strain S1133 (Vineland Laboratories, Vineland, NJ) was first adapted to grow in primary chicken embryo fibroblasts and then plaque-purified [16], which was obtained from Dr. L. H. Lee. DF-1 cells (2×10^6^ cells) were seeded onto 10-cm^2^ tissue culture dishes (Nunc, Rochester, NY) and grown until the monolayer was 70–80% confluent. The cells were then washed with phosphate-buffered saline (PBS) and infected with ARV at a multiplicity of infection (MOI) of 5 [17] or mock-infected with DMEM for 2 h at 37°C. The inoculum was then removed and the cells were washed and further incubated in maintenance medium (DMEM supplemented with 2% fetal bovine serum) for 24, 48, and 72 h for subsequent analysis.

### Immunofluorescence

To visualize changes in cell morphology upon ARV infection, indirect immunofluorescence was used as previously described [18] with some modifications. DF-1 cells were seeded at a density of 5×10^4^ cells/well on coverslips in 24-well culture plates (ThermoFisher Scientific, New York, NY) and incubated overnight at 37°C. The cells were infected for 2 h with ARV (MOI, 5) and cultured for 24, 48, and 72 h, or were mock-infected with DMEM and cultured for 72 h. Following incubation, cells were fixed with PBS containing 4% (v/v) paraformaldehyde for 25 min, washed with PBS, and permeated in PBS containing 0.2% (v/v) Triton X-100 for 10 min. The permeated cells were washed with PBS and further incubated with a mixture of rabbit anti-ARV (dilution 1∶500) and mouse anti-F-actin (dilution 1∶1000) for 1 h at 37°C. After washing with PBS, cells were incubated with a mixture of TRITC-conjugated goat anti-rabbit IgG (dilution 1∶1000) and FITC-conjugated goat anti-mouse IgG (dilution 1∶1000) for 1 h at 37°C. Coverslips were then washed three times with PBS and three times with double-distilled H_2_O before mounting on glass slides with Vectashield mounting medium (Vector Laboratories, Burlingame, CA). Cells were imaged using a Zeiss Axiovert 200M fluorescence microscope (Carl Zeiss, Mainz, Germany). Images were exported using the Zeiss AxioVision 4.0 software. Triplicate slides were prepared and analysed for each treatment.

### Sample preparation for proteomic analysis

Mock-infected cells at 72 h and ARV-infected cells at 24, 48, and 72 h post-infection (PI) were washed with PBS and lysed in buffer containing 4% (w/v) 3-[(3-cholamidopropyl)dimethylammonio]-1-propanesulfonate, 7 M urea, 2 M thiourea, 10 mM Tris-HCl, and 1 mM EDTA (pH 8.3). Cell lysates were homogenized by aspirating with a 25-G needle ten times, and the insoluble portion was removed by centrifugation at 10,000× *g* for 30 min at 4°C. Protein concentrations were determined using a Bradford protein assay (Bio-Rad Laboratories, Richmond, CA).

### 2D-DIGE and gel image analysis

**Figure 1:**
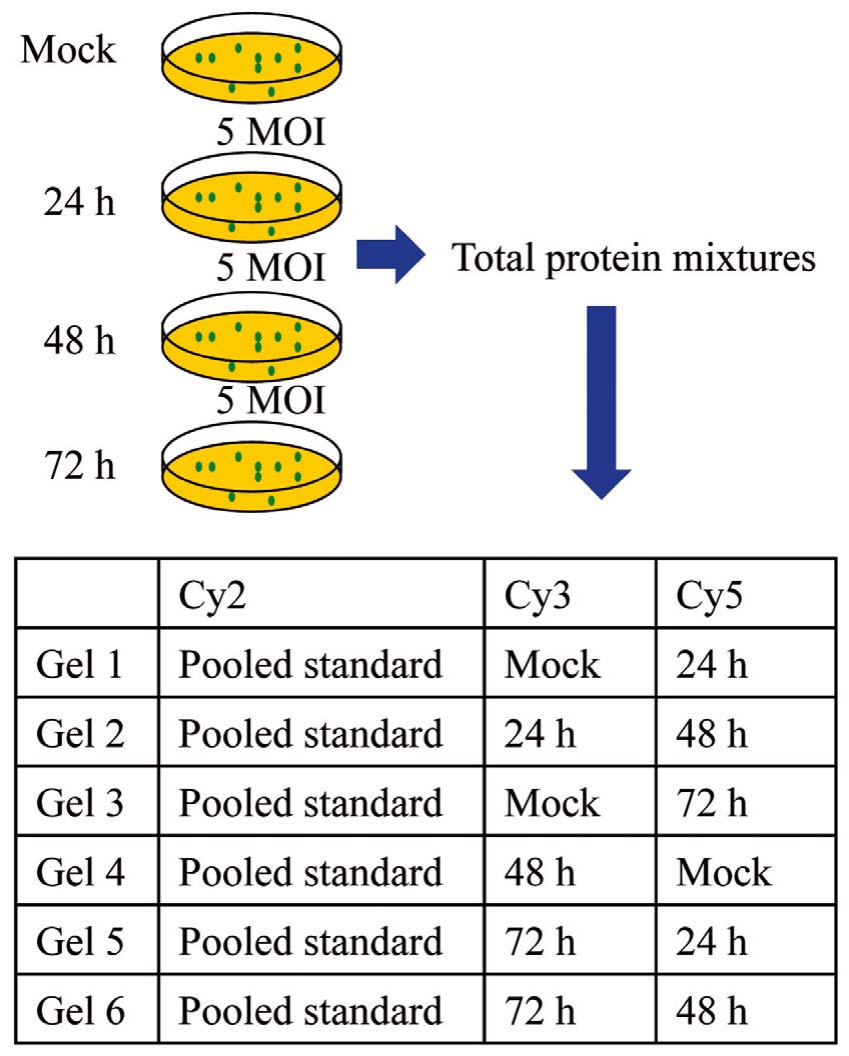
Overview of 2D-DIGE approach. Protein extracts were prepared from mock-infected DF-1 cells at 72 h post-infection (control) and ARV-infected DF-1 cells (5 MOI) at 24, 48, and 72 h post-infection. Mixed samples containing 50 µg each of Cy2-labeled pooled protein standard, Cy3- or Cy5-labeled proteins from ARV-infected cells, and Cy3- or Cy5-labeled proteins from mock-infected cells were analyzed in the indicated combinations, and each was performed in triplicate.

The experimental design for 2D-DIGE and the strategy for labeling protein samples with cyanine dyes are presented in Figure 1. Briefly, protein samples from mock-infected and AVR-infected cells were labeled with 375 pmol *N*-hydroxyl succinimidyl ester derivatives of fluorescent cyanine dye Cy3 or Cy5 (GE Healthcare) [19], [20], and pooled (50 µg each) in various combinations as indicated in Figure 1. To assist image matching and cross-gel statistical comparison, a pooled internal standard containing equal amounts of the two protein samples in 2D gel 1 was labeled with Cy2 (2.5 pmol/µg protein), and 50 µg was combined with the control and ARV-infected protein samples, giving a total of 150 µg protein on all gels (Fig. 1). The dye-labeling reactions were carried out in the dark on ice for 30 min and quenched with a 20-fold molar excess of free l-lysine (relative to dye) for 10 min. All combinations were prepared and analyzed in triplicate.

Mixed samples were reduced with dithiothreitol for 10 min. Immobilized non-linear pH gradient (IPG) strips (pH 3–10, 24 cm, GE Healthcare) were rehydrated with reduced samples in the dark at room temperature overnight. First-dimensional isoelectric focusing was performed at 20°C on a Multiphor II electrophoresis system (GE Healthcare) at 62.5 kVh. The strips were incubated in equilibration buffer containing 6 M urea, 30% (v/v) glycerol, 1% SDS, 100 mM Tris-HCl (pH 8.8), and 65 mM dithiothreitol for 15 min, followed by incubation in the same buffer containing 240 mM iodoacetamide for an additional 15 min. The equilibrated IPG strips were transferred onto 26×20−cm 12.5% polyacrylamide gels and run in an Ettan DALT*twelve* system (GE Healthcare) at 4 W per gel at 10°C until the bromophenol blue dye front had migrated to the bottom of the gels. Each 2D gel was run in triplicate. The 2D gels were scanned at the appropriate wavelengths for Cy2, Cy3, and Cy5 using an Ettan DIGE Imager (GE Healthcare), and the scans analyzed using DeCyder 2-D Differential Analysis Software v7.0 (GE Healthcare) to detect, normalize, and quantify the protein features in the images. Spots displaying a ≥1.5-fold increase or decrease in intensity of the AVR-infected samples relative to the mock-infected samples (with *p*<0.05) were selected for protein identification.

### Protein staining

Coomassie Blue staining was used to visualize CyDye-labeled proteins in the 2D gels. Gels were fixed in 2% (v/v) phosphoric acid, 30% (v/v) ethanol overnight at room temperature. The gels were then washed with double-distilled H_2_O three times (30 min each) and incubated in a solution of 3% (v/v) phosphoric acid, 17% ammonium sulfate, and 34% (v/v) methanol for 1 h. Gels were stained in Coomassie Brilliant Blue G-250 (colloidal, 0.5 g/l; ThermoFisher Scientific, New York, NY) for 5–7 days. The gels were not destained prior to analysis, which was performed using an ImageScanner III densitometer (GE Healthcare).

### In-gel digestion

Protein spots of interest were cut from the stained gels, washed three times in 50% (v/v) acetonitrile, and dried in a SpeedVac vacuum concentrator. Excised spots were then reduced with 10 mM dithiothreitol in 5 mM ammonium bicarbonate (pH 8.0) for 45 min at 50°C and alkylated with 50 mM iodoacetamide in 5 mM ammonium bicarbonate for 1 h at room temperature in the dark. The gel pieces were then washed three times in 50% (v/v) acetonitrile and dried in the SpeedVac. The dried gel pieces were rehydrated with 50 ng modified trypsin (Promega, Madison, WI) in 10 µl of 5 mM ammonium bicarbonate and digested for 16 h at 37°C. Supernatants were collected and peptides were extracted twice with 5% (v/v) trifluoroacetic acid in 50% (v/v) acetonitrile. The supernatants containing extracted peptides were pooled, dried under vacuum, resuspended in 5 µl double-distilled H_2_O, and stored at −20°C prior to MS analysis.

### Protein identification by MALDI-TOF MS

MALDI-TOF-MS-generated peptide mass fingerprinting was utilized for protein identification [20]. Briefly, 0.5 µl trypsin-digested protein sample was mixed with 0.5 µl of matrix solution containing 1 mg/ml α-cyano-4-hydroxycinammic acid in 50% (v/v) acetonitrile, 0.1% (v/v) trifluoroacetic acid and then spotted onto an AnchorChip target plate (Bruker Daltonics, Fremont, CA) and dried. Peptide mass fingerprints were obtained using an Autoflex III mass spectrometer (Bruker Daltonics) in reflector mode. The Sophisticated Numerical Annotation Procedure (SNAP) algorithm was used for spectrum annotation using the following detailed metrics: peak detection algorithm, SNAP; signal-to-noise threshold, 25; relative intensity threshold, 0%; minimum-intensity threshold, 0; maximal number of peaks, 50; quality factor threshold, 1000; SNAP average composition, averaging; baseline subtraction, median flatness, 0.8; median level, 0.5. The spectrometer was calibrated with a peptide calibration standard (Bruker Daltonics), and internal calibration was performed using trypsin autolysis peaks at *m*/*z* 842.51 and 2211.10. Peaks in the mass range of *m*/*z* 800 to 3000 were used to generate a peptide mass fingerprint that was searched against the updated Swiss-Prot/TrEMBL database (Dec 11, 2013 release; 49,243,530 entries http://www.uniprot.org/uniprot/) using Mascot software v2.3.00 (Matrix Science, London, UK) with the following parameters: All; mammals; tryptic digest with a maximum of one missed cleavage; mass tolerance, 50 ppm. Identification was accepted based on significant Mascot MOWSE scores (*p*<0.05), spectrum annotation, and observed versus expected molecular weight and isoelectric point (pI) in the 2D gels.

### Immunoblotting

Mock-infected and ARV-infected cells were lysed with buffer containing 50 mM HEPES (pH 7.4), 150 mM NaCl, 1% NP-40, 1 mM EDTA, 2 mM sodium orthovanadate, 100 µg/ml 4-(2-aminoethyl) benzenesulfonyl fluoride hydrochloride, 17 µg/ml aprotinin, 1 µg/ml leupeptin, 1 µg/ml pepstatin, 5 µM fenvalerate, 5 µM potassium bisperoxo (1, 10-phenanthroline) oxovanadate, and 1 µM okadaic acid prior to protein quantification by Bradford protein assay (Bio-Rad Laboratories). Protein samples (30 µg) were diluted (1∶1) in Laemmli sample buffer [21] (50 mM Tris-HCl, 10% (v/v) glycerol, 2% SDS, 0.01% bromophenol blue, pH 6.8) and separated by SDS-polyacrylamide gel electrophoresis (SDS-PAGE; 15%). Separated proteins were electroblotted onto 0.45-µm Immobilon P membranes (Millipore, Billerica, MA), which were then blocked with 5% (w/v) skim milk in TBST (50 mM Tris, pH 8.0, 150 mM NaCl, 0.1% [v/v] Tween-20) for 1 h. Membranes were incubated with individual primary antibodies in TBST containing 0.02% (w/v) sodium azide at 4°C for 2 h. Membranes were washed three times with TBST, then incubated with horseradish peroxidase−conjugated goat anti-mouse IgG or goat anti-rabbit IgG at 4°C for 1 h, and visualized using enhanced chemiluminescence (GE Healthcare).

## Results

### Immunofluorescence analysis of cell morphology

**Figure 2:**
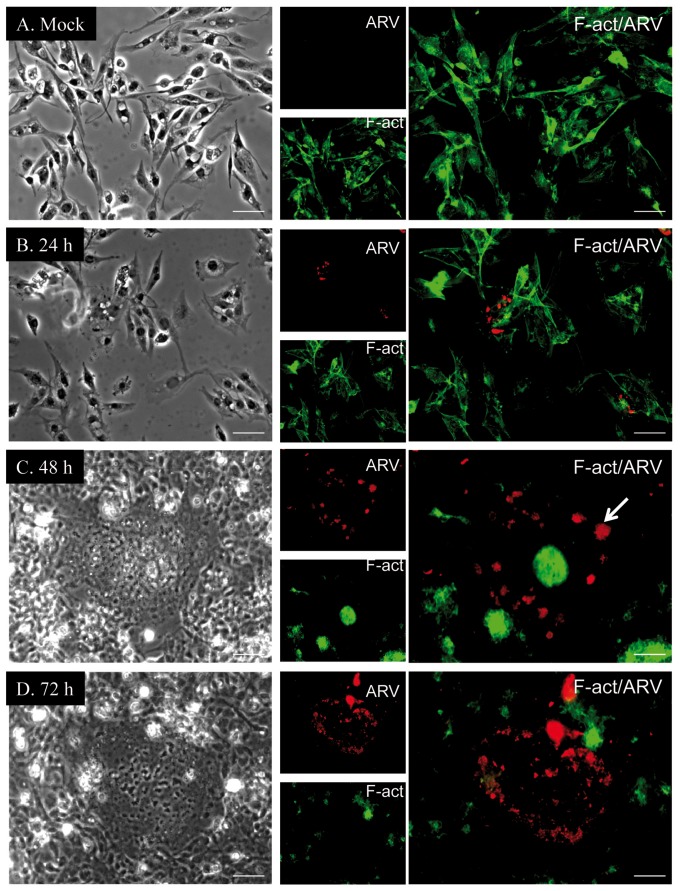
Time-course analysis of ARV infection and cell morphology by immunofluorescence. In ARV- (B−D) or mock- (A, control) infected DF-1 cells, F-actin was visualized using mouse monoclonal anti-F-actin (green), and viral proteins were detected using mouse polyclonal anti-ARV (red). ARV-infected cells were analyzed at (B) 24, (C) 48, and (D) 72 h post-infection. All experiments were performed in triplicate. Each set of three fields was taken using the same exposure conditions and each image is representative of six different fields examined. Brightfield microscopy images are shown in the left panels. Scale bar = 10 µm.

We utilized an indirect immunofluorescence assay to monitor morphological changes and viral proliferation in DF-1 cells at different time points after ARV infection (Fig. 2). At 24 h PI, only slight viral protein signals were detected in ARV-infected cells (Fig. 2B). The majority of stained cells maintained normal morphology, and their *cytoskeletal* structure were intact, as determined by the distribution of F-actin. At 48 h PI, the morphology of ARV-infected cells was clearly disrupted, and F-actin staining was condensed into discrete foci, indicating disassembly of actin filaments (Fig. 2C). Syncytium formation was also observed in ARV-infected cells at 48 h (arrow in Fig. 2C). At 72 h PI, >90% of the cells were infected by ARV, and almost all F-actin in the infected cells was disassembled (Fig. 2D).

### 2D-DIGE analysis of ARV- and mock-infected DF-1 cells

**Figure 3:**
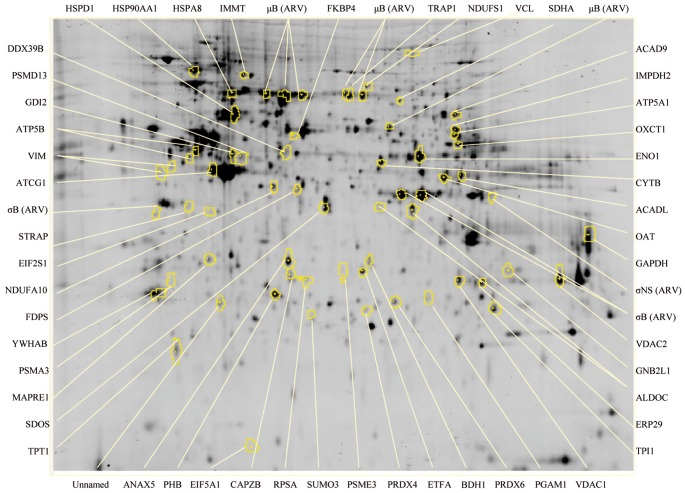
Representative 2D-DIGE map of differentially expressed proteins in ARV-infected DF-1 cells. Equal amounts of ARV- and mock-infected cell lysates were labeled with cyanine dyes (see Materials and Methods and Fig. 1) and separated using IPG strips followed by polyacrylamide gel electrophoresis. Circles indicate successfully identified protein spots, as indicated by protein names.

To investigate changes in the abundance of host proteins during ARV infection, comparative proteomic analysis of ARV- and mock-infected DF-1 cells was performed by 2D-DIGE. More than 1000 protein spots were detected on the gels, with the majority of differential protein expression observed at 72 h PI (Fig. 3). A representative of gel 3 (control vs. 72 h PI) is shown. To minimize gel-to-gel variability, only protein spots that appeared in three replicate gels were quantified for statistical analysis. Additionally, spots displaying a ≥1.5-fold difference in expression with a *t*-test *p*<0.05 were visually confirmed prior to protein identification.

### MALDI-TOF-MS identification of differentially expressed proteins

**Figure 4:**
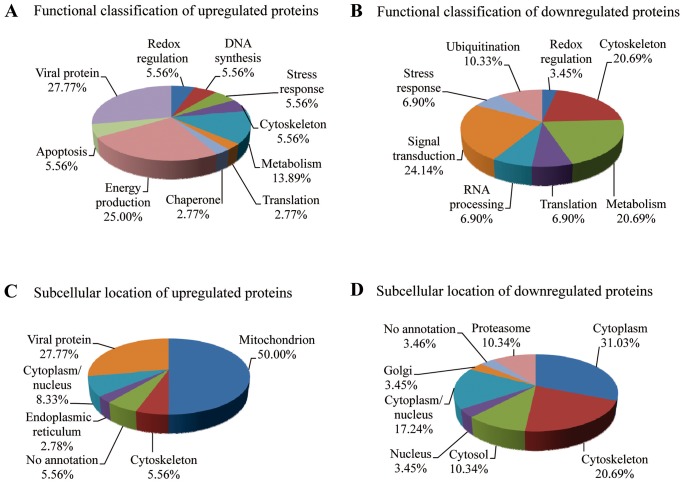
Functional classification and subcellular locations of differentially expressed proteins in ARV-infected DF-1 cells. (A and B) functional classifications and (C and D) subcellular locations of upregulated and downregulated proteins in ARV-infected DF-1 cells identified by 2D-DIGE/MALDI-TOF MS.

To determine the identity of the differentially expressed proteins (≥1.5-fold change by 2D-DIGE analysis) in ARV-infected DF-1 cells, protein spots were excised from the gels, trypsinized, and analyzed by MALDI-TOF MS. Of the 98 protein spots, 65 were successfully identified (Table S1). The function and subcellular localization of these proteins was determined according to matches in the Swiss-Prot/TrEMBL protein database (Fig. 4). The percentages of up- and downregulated proteins were 55.38% and 44.62%, respectively. The upregulated proteins were mostly viral proteins (27.77%), or were cellular proteins involved in energy production (25.00%) and metabolism (13.89%; Fig. 4A), whereas the downregulated proteins were mainly cellular proteins involved in signal transduction (24.14%), metabolism (20.69%), and the ubiquitin-proteasome pathway (UPP; 10.33%; Fig. 4B). The upregulated proteins were mostly located in the mitochondria (50.00%), cytoplasm (27.77%), and cytoplasm/nucleus (8.33%; Fig. 4C). The downregulated proteins were mostly located in the cytoplasm (31.03%), cytoskeleton (20.69%), and cytoplasm/nucleus (17.24%; Fig. 4D).

Comparing the ARV-infected cells at different time points, we identified the three proteins that were upregulated at 24 h PI as ARV proteins μB, μNS, and σB. The cytoskeletal protein vimentin was the only downregulated protein identified at 24 h PI. At 48 h PI, four additional proteins were upregulated, including one involved in energy production (ATP synthase subunit β), one involved in signal transduction (TNF receptor-associated protein 1), and two viral proteins (σC and σNS). Vimentin was still the only downregulated protein identified at 48 h PI. At 72 h PI, an additional 51 proteins were differentially expressed, 25 of which were upregulated, and 26 of which were downregulated. Hence, the majority of protein expression changes were induced between 48 and 72 hr PI. Notably, all of the identified proteins involved in signal transduction, RNA processing, and the UPP were downregulated, whereas proteins involved in DNA synthesis, apoptosis, and energy production were upregulated. In summary, a schematic overview of pathways and the cellular proteins that are found significantly regulated during ARV infection of DF-1 cells is shown in Figure S1.

### Immunoblot confirmation of representative proteins in ARV-infected DF-1 cells

**Figure 5:**
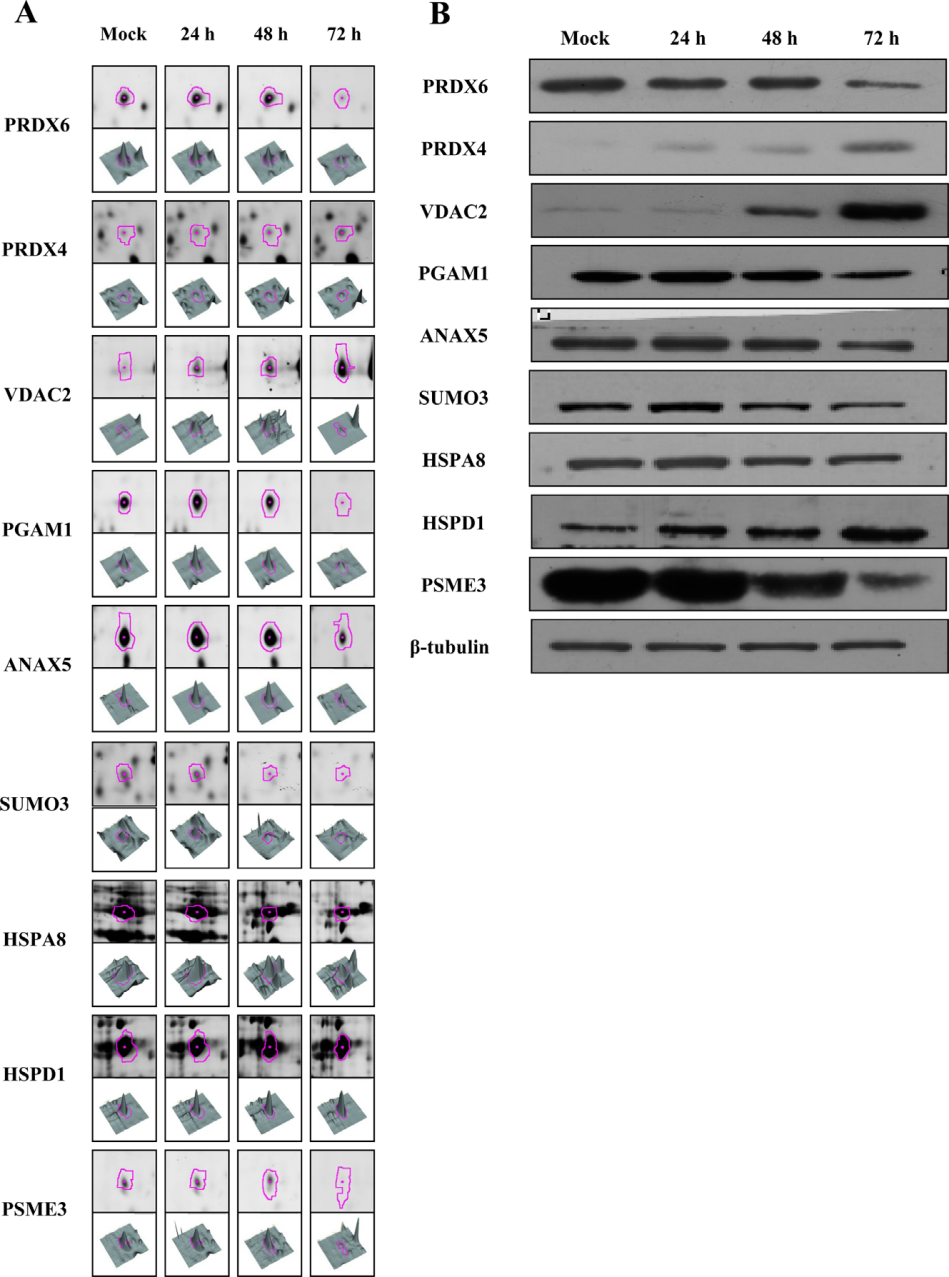
Representative western blot for verification of selected differentially expressed proteins in ARV-infected DF-1 cells. (A) 2D-DIGE spots (pink outline) and 3D representations of spot intensities for representative upregulated proteins (PRDX4, VDAC2, and HSPD1) and downregulated proteins (PRDX6, PGAM1, ANXA5, SUMO3, HSPA8, and PSME3). (B) Equal amounts of total cell lysates from mock-infected (72 h PI) and ARV-infected DF-1 cells at 24, 48, and 72 h PI were subjected to SDS-PAGE and immunoblot analysis using antibodies against the indicated proteins PRDX6, PRDX4, VDAC2, PGAM1, ANXA5, SUMO3, HSPA8, HSPD1, and PSME3. β-Tubulin was used as an internal control and for normalization.

To confirm the MALDI-TOF MS results, ten differentially expressed proteins (PRDX6, PRDX4, VDAC2, PGAM1, ANAX5, SUMO3, HSPA8, HSPD1, PSME3, and PSMD13) were chosen for immunoblot analysis, along with β-tubulin as an internal control for normalization. Equal amounts of cell lysates from ARV-infected cells at 24, 48, and 72 h PI and mock-infected cells at 72 h PI were used in western blot analysis with specific polyclonal antibodies to these proteins. The presence of PRDX6, PRDX4, VDAC2, PGAM1, ANAX5, SUMO3, HSPA8, HSPD1, PSME3, and β-tubulin was confirmed using individual polyclonal antibodies (Fig. 5), but PSMD13 was not detected by their corresponding polyclonal antibodies (data not shown). PRDX4, VDAC2, and HSPD1 were more highly expressed in ARV-infected DF-1 cells than in the mock-infected control, whereas PRDX6, PGAM1, ANXA5, SUMO3, HSPA8, and PSME3 were expressed at lower levels than in the control. These results were consistent with the expression changes seen in the 2D-DIGE analysis.

## Discussion

Although many studies have investigated the structure, function, and protein interactions of ARV [22], [23] as well as the relationships between ARV and host cells [24]–[27], comprehensive changes in the host cell proteome in response to ARV infection have not been explored. To our knowledge, the current study is the first to use 2D-DIGE coupled with MALDI-TOF MS to identify differentially expressed protein profiles in ARV-infected cells, which may be crucial in ARV pathogenesis. Interestingly, energy production, DNA synthesis, and protein biosynthesis appear to have been upregulated in ARV-infected cells, but RNA processing, signal transduction, and the UPP were downregulated.

### Changes in host cytoskeleton after ARV infection

The cytoskeleton is involved in the entry, replication, and exit of many viruses, such as herpes viruses [28], human immunodeficiency virus (HIV) [29] and mammalian reovirus [30], and many viruses also utilize the host cytoskeleton to promote viral infection [31]. In this study, actin (cytoplasmic type 5) and syndesmos (SDOS) were upregulated in ARV-infected cells, whereas vinculin, vimentin, microtubule-associated protein RP/EB family member 1 (MAPRE1), F-actin-capping protein subunit β isoform 1 and 2 (CAPZB), and translationally controlled tumor protein (TCTP) were downregulated. ARV genomic segment S4 encodes a fusion-associated small transmembrane protein that mediates cell-cell fusion, and this cell fusion activity requires active cellular actin remodeling [32]. Similarly, our immunofluorescence analysis showed that the cellular actin cytoskeleton of DF-1 cells was dramatically restructured/disordered at 48 and 72 h after ARV infection (Fig. 2). Thus, the rearrangement of actin filament structure may play an important role in facilitating ARV infection processes.

Chicken SDOS is ubiquitously expressed and is involved in cytoskeletal assembly [33]. Overexpression of chicken SDOS in NIH 3T3 cells enhances cell spreading and formation of actin stress fibers and focal contacts [33]. We observed a >17-fold increase in SDOS expression in ARV-infected cells at 72 h PI, suggesting that ARV infection-induced SDOS expression may be responsible for the observed cytoskeletal reorganization. MAPRE1 can interact with the C terminus of adenomatous polyposis coli protein to promote microtubule polymerization [34]. Downregulation of MAPRE1 has also been observed in cells infected with HIV-1 and infectious bursal disease virus (IBDV) [35]. Chicken CAPZB is known to regulate the growth of actin filaments by capping the barbed end [36], and thus it may also have a role in cytoskeletal reorganization during viral infection. TCTP binds tubulin [37], acts as an anti-apoptotic factor [38], and is downregulated in herpes simplex virus-1 infected cells [39].

### Energy production and metabolism

Ours is the first study to show the dynamic effects of ARV on host cell metabolism. We found that ARV infection leads to metabolic changes in infected DF-1 cells that appear to favor an increase in energy production from amino acid and fatty acid catabolism, oxidative phosphorylation, and the tricarboxylic acid cycle. These results are similar to what has been observed in HIV-infected T cells [40], although the metabolism-related proteins involved are not identical. Upregulation of energy-associated proteins may promote ARV replication and other energy-dependent biological processes [41]. Conversely, several proteins involved in the glycolysis pathway were downregulated in ARV-infected DF-1 cells, similar to observations in HIV-, IBDV- [12], and chikungunya virus-infected host cells [42].

Previous studies have proposed that ARV triggers autophagy in infected host cells through activation of the p53 signaling pathway [9]. Autophagy is also observed in dengue virus infection, where it alters cellular lipid metabolism, thereby facilitating viral replication. [43]. We also observed ARV-related upregulation of mitochondrial proteins involved in lipid metabolism. It will be interesting to examine whether ARV-induced autophagy regulates host cell lipid metabolism. p53 regulates autophagy and glucose metabolism via the TP53-induced glycolysis and apoptosis regulator (TIGAR), and the crucial functions of TIGAR are to block glycolysis, restrict production of reactive oxygen species (ROS), and limit apoptosis [44]. Therefore, it will also be important to determine whether p53 and TIGAR contribute to the observed changes in metabolic pathways observed in ARV-infected host cells.

A key glycolytic enzyme, **glyceraldehyde-3-phosphate dehydrogenase (GAPDH)**, was upregulated in ARV-infected cells. GAPDH participates in oxidation of d-glyceraldehyde 3-phosphate to 3-phospho-d-glyceroyl phosphate and reduction of NAD+ to NADH. An earlier study found a gradual increase in ROS during ARV S1133 infection of DF1 cells [10]. This suggests that upregulation of GAPDH may not only promote energy production but may also serve to overcome the oxidative stress induced during ARV infection. GAPDH also contributes to various other non-glycolytic processes, including nuclear membrane fusion [45], microtubule binding [46], RNA transport, and apoptosis [47]. GAPDH may also play a direct role in the viral life cycle. GAPDH has been shown to physically interact with RNA from Japanese encephalitis [48], hepatitis A [49] and parainfluenza viruses [50]. These findings raise the interesting question of whether GAPDH is involved in these non-glycolytic activities during ARV infection.

### RNA processing and translation machinery

Viral hijacking of the host cell mRNA processing and translation apparatus is critical for replication of many viruses [12], [42]. In this study, we identified changes in three translation-related proteins in ARV-infected DF-1 cells, including downregulation of two eukaryotic translation initiation factors (EIF2S and EIF5A1) and upregulation of 40S ribosomal protein SA (RPSA). This result suggests that ARV turns off the host cell translation apparatus and initiates viral translation, consistent with the recent report that ARV non-structural protein p17 inactivation of host cell protein translation involves eIF2α and eEF2 [25]. Moreover, upregulation of RPSA has also been observed in severe acute respiratory syndrome coronavirus- and IBDV-infected cells, suggesting that it may play a role in the viral multiplication process in host cells. Notably, EIF5A1 has also been shown to induce apoptosis through activation of the intrinsic mitochondrial pathway [51] and to play an important role in mRNA turnover [52].

### The UPP

The UPP is the primary intracellular protein degradation system and plays important roles in apoptosis, cell cycle control, signal transduction, and transcriptional regulation [53]. The host UPP also appears to be required for virion development, progeny release, and effective replication of some viruses [54]. In our study, three UPP-related proteins were downregulated by ARV infection: proteasome activator complex subunit (PSME3), proteasome (prosome, macropain) subunit α type 3 (PSMA3), and 26S proteasome non-ATPase regulatory subunit 13 (PSMD13). ARV infection has previously been shown to cause apoptosis and enhance p53 expression [55]. In addition, PSME3 inhibits apoptosis and promotes MDM2-mediated p53 degradation [56]. The downregulation of PSME3 that we observed suggests that ARV infection likely reduces MDM2-dependent degradation of p53 in DF-1 cells and promotes apoptosis. Decreases in PSME3 have also been observed in IBDV-infected chicken embryo fibroblasts [12] and H1N1 influenza virus−infected porcine alveolar macrophages [57]. In addition, PSMA3 and PSMD13 appear to physically interact in human cells [58]. Our data suggest that ARV infection causes functional impairment of the cellular UPP to facilitate viral progeny release.

### Redox regulation

Peroxiredoxins are a ubiquitous family of antioxidant enzymes that also regulate cytokine-induced peroxide levels and thereby mediate signal transduction in mammalian cells [59]. In our study, downregulated peroxiredoxin-6 (PRDX6) and upregulated peroxiredoxin-4 (PRDX4) were observed during ARV infection. Previous study indicated that transient transfection of liver cells with PRDX6 siRNA resulted in a rise in peroxide-induced cytotoxicity by apoptosis, implying that suppression of PRDX6 promotes apoptosis [60]. Therefore, the downregulation of PRDX6 here we observed suggests that ARV infection decreases the anti-apoptotic function for PRDX6. On the other hand, upregulation of PRDX4 has been detected in respiratory syncytial virus-infected human pulmonary epithelial cells, suggesting that PRDX4 may play an important role to protect nuclear proteins against respiratory syncytial virus-induced oxidative stress [61].

### Signal transduction

In our studies, two cellular proteins associated with signal transduction were downregulated by ARV infection including annexin A5 (ANXA5) and small ubiquitin-related modifier 3 (SUMO3). ANXA5 is anticipated in many cellular processes, such as signaling transduction, membrane trafficking, blood coagulation regulation, and shielding exposed phospholipids (REF15576370). SUMOylation is a post-translational modification, which covalent conjugated with ubiquitin-like proteins named SUMO-1, -2, and -3 to target proteins (12672486). Previous study has indicated that MDM2 can promote SUMO-2 and -3 modification of P53 to both activate and repress some P53-target genes (PMC3218624). To our knowledge, decreased expression levels of ANXA5 and SUMO3 proteins have not been reported in virus-infected cells. The reason of downregulated ANXA5 and SUMO3 at late stage of ARV infection is still unclear. Our data suggest that decrease in SUMO3 reduces the conjugation of P53 and promotes apoptosis in ARV infected DF-1 cells.

### Apoptosis pathway-associated proteins

We found that one apoptosis-related protein was downregulated by ARV infection (YWHAB) and several others were upregulated (HSPD1, ERP29, PHB, IMPDH2, VDAC1, VDAC2, and viral protein σC). ARV σC is known to induce apoptosis in cultured cells [17] through p53 and the mitochondria-mediated pathway [62]. Upregulation of σC in this study suggests that it also promotes apoptosis in DF-1 cells. HSPD1 is a mitochondrial chaperonin that promotes pro-caspase-3 maturation and regulates apoptosis [63]. HSPD1 is also upregulated in cells infected with H1N1 influenza virus [57]. Moreover, the pro-apoptotic protein VDAC2 is a mitochondrial porin [64] that regulates cell permeability and appears to cause swelling of the mitochondrial matrix in Nipah virus−infected cells [65]. A viral protein from another chicken dsRNA virus, IBDV, has also been shown to induce apoptosis in DF-1 cells through interaction with VDAC2 [66], but a specific interaction between ARV proteins and VDAC2 has yet to be demonstrated. Moreover, the anti-apoptotic protein YWHAB, which was downregulated by ARV in our study, is thought to play a crucial role in the anti-viral innate immune response [67]. These ARV-induced changes in apoptosis regulating proteins are similar to those observed in HIV-infected T cells [40].

### Summary

This proteomic approach based on 2D-DIGE and MALDI-TOF MS identified 65 differentially expressed proteins in cultured chicken fibroblasts during ARV infection, nine of which were verified by immunoblotting. The proteins identified in this study may play important roles in viral pathogenesis and may become potential therapeutic targets to manage ARV infections.

## Supporting Information

Figure S1 Schematic overview of pathways and involved proteins which are found significantly regulated in ARV-infected DF-1 cells (arrows: up-regulated, bars: down-regulated). Here, the identified cellular proteins (blue), named in accordance with Table S1, and their biological functions (green) are displayed.
(DOCX)

Table S1 Differentially expressed proteins in ARV-infected DF-1 cells identified by MALDI-TOF MS.
(DOCX)
