# Supplementary material for: Proteomics Analysis of the DF-1 Chicken Fibroblasts Infected with Avian Reovirus Strain S1133
Source: PLoS One. 2014 Mar 25;9(3):e92154. doi: 10.1371/journal.pone.0092154 (PMC3965424; doi:10.1371/journal.pone.0092154)
Supplement: Figure S1 — Schematic overview of pathways and involved proteins which are found significantly regulated in ARV-infected DF-1 cells (arrows: up-regulated, bars: down-regulated). Here, the identified cellular proteins (blue), named in accordance with Table S1, and their biological functions (green) are displayed. (DOCX) [file pone.0092154.s001.docx]

Figure S1


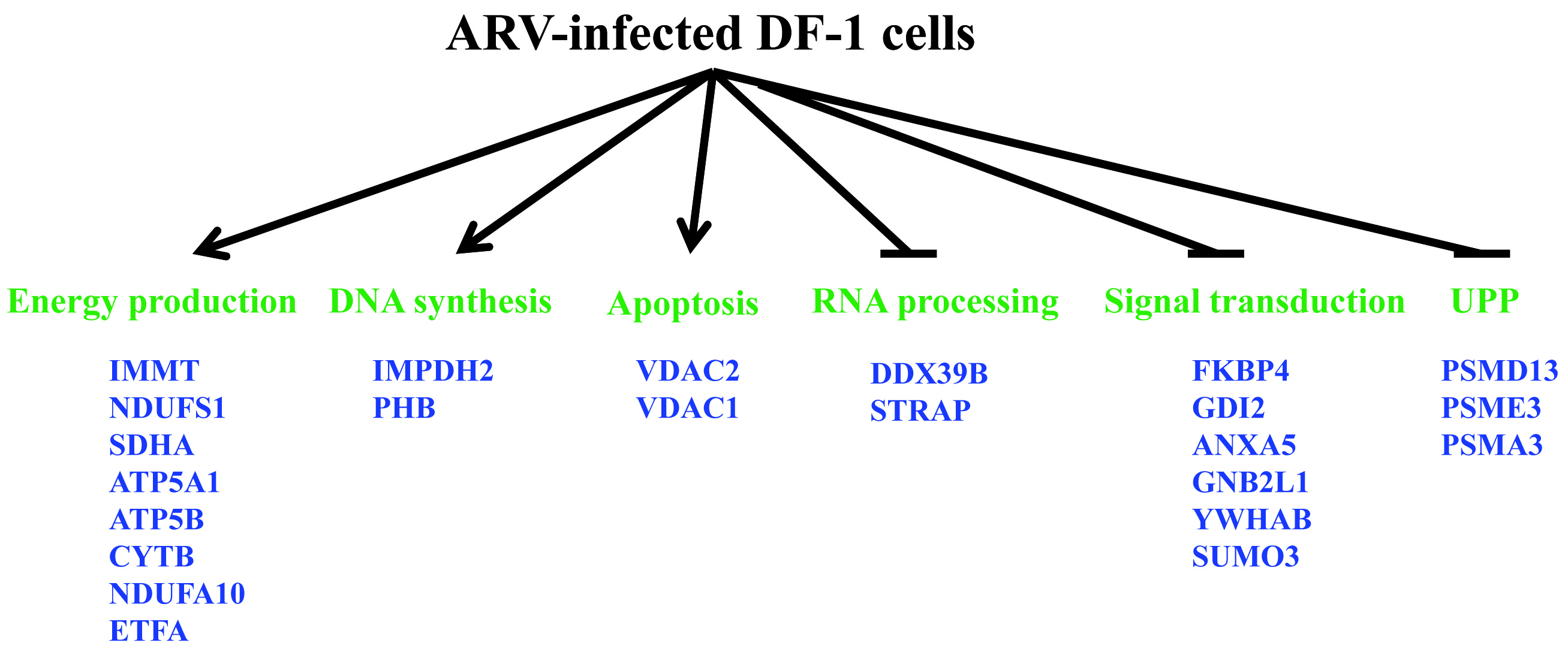


**Figure S1**. Schematic overview of pathways and involved proteins which are found significantly regulated in ARV-infected DF-1 cells (arrows: up-regulated, bars: down-regulated). Here, the identified cellular proteins (blue), named in accordance with Table S1, and their biological functions (green) are displayed.
