# Supplementary material for: Proteomics Analysis of the DF-1 Chicken Fibroblasts Infected with Avian Reovirus Strain S1133
Source: PLoS One. 2014 Mar 25;9(3):e92154. doi: 10.1371/journal.pone.0092154 (PMC3965424; doi:10.1371/journal.pone.0092154)
Supplement: Table S1 — Differentially expressed proteins in ARV-infected DF-1 cells identified by MALDI-TOF MS. (DOCX) [file pone.0092154.s002.docx]

**Table S1.** Differentially expressed proteins in ARV-infected DF-1 cells identified by MALDI-TOF MS.

| **Spot number^a^** | **Accession number^b^** | **Protein name** | **Abbreviation** | **MW** | **p*I*** | **Coverage^c^** | **Score** | **No. peptides match/supplied** | **Peptide sequence** | **24 h/Mock^d^** | **48 h/Mock^d^** | **72 h/Mock^d^** | **Location^e^** | **Function^e^** |
| --- | --- | --- | --- | --- | --- | --- | --- | --- | --- | --- | --- | --- | --- | --- |
| 1745 | gi\|68564977 | d-β-Hydroxybutyrate dehydrogenase, mitochondrial | BDH1 | 38611 | 8.37 | 22% | 71/69 | 7/25 | KYFDEQVSK,FGVEAFSDCLR | −1.24 | 1.08 | 1.87 | Mitochondrion | Redox regulation |

| 1937 | gi\|82233783 | Peroxiredoxin-6 | PRDX6 | 25075 | 5.72 | 37% | 112/69 | 10/33 | LSILYPATTGR,DFTPVCTTELGR | 1.1 | −1.03 | −2.11 | Cytoplasm | Redox regulation |
| --- | --- | --- | --- | --- | --- | --- | --- | --- | --- | --- | --- | --- | --- | --- |

| 1957 | gi\|118084003 | Similar to MGC82793 protein isoform 1= peroxiredoxin 4 | PRDX4 | 30108 | 5.94 | 38% | 120/72 | 9/16 | VPVTDHSLHLSQAK,GLFIIDDKR | −1.07 | 1.06 | 1.79 | Mitochondrion | Redox regulation |
| --- | --- | --- | --- | --- | --- | --- | --- | --- | --- | --- | --- | --- | --- | --- |

| 1804 | gi\|46048903 | Voltage-dependent anion channel 2 | VDAC2 | 30293 | 8.61 | 45% | 112/72 | 10/35 | YKLDSTASISAK,NNFSVGYK | −1.01 | 1.16 | 2.99 | Mitochondrion | Apoptosis |
| --- | --- | --- | --- | --- | --- | --- | --- | --- | --- | --- | --- | --- | --- | --- |

| 1820 | gi\|76443696 | Voltage-dependent anion channel 1 | VDAC1 | 30744 | 6.85 | 48% | 138/72 | 10/37 | LTFDSSFSPNTGK,VTQSNFAVGYK | −1.17 | 1.04 | 2.26 | Mitochondrion | Apoptosis |
| --- | --- | --- | --- | --- | --- | --- | --- | --- | --- | --- | --- | --- | --- | --- |
| 176 | gi\|50403716 | Vinculin | VCL | 125052 | 5.43 | 14% | 68/61 | 13/35 | GILSGTSDLLLTFDEAEVR,DPNAPPGDAGEQAIR | −1.07 | −1.21 | −2.98 | Cytoskeleton | Cytoskeleton |

| 1145 | gi\|138534 | Vimentin | VIM | 53167 | 5.09 | 21% | 92/62 | 10/38 | MFGGGSRPSSGTR,EMEENFAVEAANYQDTIGR | −2.73 | −2.52 | −1.85 | Cytoskeleton | Cytoskeleton |
| --- | --- | --- | --- | --- | --- | --- | --- | --- | --- | --- | --- | --- | --- | --- |

| 1171 | gi\|138535 | Vimentin | VIM | 53167 | 5.09 | 25% | 72/69 | 12/45 | INMPIPTFASLNLR,FADLSEAANR | −3.52 | −3.51 | −2.43 | Cytoskeleton | Cytoskeleton |
| --- | --- | --- | --- | --- | --- | --- | --- | --- | --- | --- | --- | --- | --- | --- |

| 1206 | gi\|1703121 | Actin, cytoplasmic type 5 | ACTG1 | 42151 | 5.3 | 35% | 87/69 | 11/35 | AGFAGDDAPR,QEYDESGPSIVHR | 1.02 | 1.02 | 1.57 | Cytoskeleton | Cytoskeleton |
| --- | --- | --- | --- | --- | --- | --- | --- | --- | --- | --- | --- | --- | --- | --- |

| 1459 | gi\|68566062 | Protein syndesmos | SDOS | 33446 | 5.74 | 36% | 112/69 | 15/42 | VVAHLYAR,DHGLEVMGMVR | −1.46 | 1.25 | 17.53 | Cytoskeleton | Cytoskeleton |
| --- | --- | --- | --- | --- | --- | --- | --- | --- | --- | --- | --- | --- | --- | --- |

| 1722 | gi\|60390179 | Microtubule-associated protein RP/EB family member 1 | MAPRE1 | 29296 | 5.04 | 55% | 164/69 | 13/34 | FQDNFEFVQWFK,ERDFYFGK | 1.04 | −1.04 | −2.13 | Cytoskeleton | Cytoskeleton |
| --- | --- | --- | --- | --- | --- | --- | --- | --- | --- | --- | --- | --- | --- | --- |

| 1807 | gi\|115597 | F-actin-capping protein subunit β isoforms 1 and 2 | CAPZB | 31573 | 5.36 | 48% | 147/69 | 15/38 | LEVEANNAFDQYR,RLPPQQIEK | −1.08 | 1.01 | −1.51 | Cytoskeleton | Cytoskeleton |
| --- | --- | --- | --- | --- | --- | --- | --- | --- | --- | --- | --- | --- | --- | --- |
| 2074 | gi\|1174624 | Translationally controlled tumor protein | TPT1 | 19689 | 4.9 | 45% | 119/69 | 9/26 | DCISQDEMFSDIYK,LEEHKPER | 1.27 | −1.12 | −1.61 | Cytoskeleton | Cytoskeleton |

| 412 | gi\|57530041 | Inner membrane protein, mitochondrial (mitofilin) | IMMT | 79543 | 5.72 | 15% | 94/72 | 7/27 | ASCSDNAFTEALTAALPQESLTR,IVAQYHELVATAR | −1.23 | 1.06 | 1.69 | Mitochondrion | Energy production |
| --- | --- | --- | --- | --- | --- | --- | --- | --- | --- | --- | --- | --- | --- | --- |

| 532 | gi\|57529754 | NADH dehydrogenase (ubiquinone) Fe-S protein 1, 75 kDa (NADH-coenzyme Q reductase) | NDUFS1 | 80609 | 6.5 | 24% | 101/72 | 12/31 | KVESIDVLDAVGSNIVVSTR,TKSGVGSDWK | −1.09 | 1.06 | 1.64 | Mitochondrion | Energy production |
| --- | --- | --- | --- | --- | --- | --- | --- | --- | --- | --- | --- | --- | --- | --- |

| 659 | gi\|205831552 | Succinate dehydrogenase [ubiquinone] flavoprotein subunit, mitochondrial | SDHA | 74025 | 6.65 | 28% | 142/69 | 14/27 | RPFEEHWR,AFGGQSLQFGK | −1.05 | 1.18 | 1.87 | Mitochondrion | Energy production |
| --- | --- | --- | --- | --- | --- | --- | --- | --- | --- | --- | --- | --- | --- | --- |

| 1007 | gi\|45383567 | ATP synthase, H^+^ transporting, mitochondrial F1 complex, alpha subunit | ATP5A1 | 60263 | 9.29 | 28% | 88/72 | 11/47 | EVAAFAQFGSDLDAATQQLLNR,AVDSLVPIGR | −1.06 | 1.24 | 2.2 | Mitochondrion | Energy production |
| --- | --- | --- | --- | --- | --- | --- | --- | --- | --- | --- | --- | --- | --- | --- |

| 1041 | gi\|82082513 | ATP synthase subunit beta, mitochondrial | ATP5B | 56650 | 5.59 | 57% | 228/69 | 30/60 | VVDLLAPYAK,FTQAGSEVSALLGR | −1.06 | 1.21 | 2.74 | Mitochondrion | Energy production |
| --- | --- | --- | --- | --- | --- | --- | --- | --- | --- | --- | --- | --- | --- | --- |

| 1095 | gi\|71897237 | ATP synthase, H+ transporting, mitochondrial F1 complex, β subunit | ATP5B | 56650 | 5.59 | 49% | 177/72 | 18/53 | FLSQPFQVAEVFTGHMGK,RAAGPSHGFLPLLLSR | −1.14 | 1.75 | 1.73 | Mitochondrion | Energy production |
| --- | --- | --- | --- | --- | --- | --- | --- | --- | --- | --- | --- | --- | --- | --- |

| 1151 | gi\|196049775 | Chicken cytochrome Bc1 complex | CYTB | 49981 | 5.95 | 35% | 141/72 | 16/42 | CPALAAVGPIEQLLDYNR,EDAVPILPR | −1.12 | 1.06 | 2.09 | Mitochondrion | Energy production |
| --- | --- | --- | --- | --- | --- | --- | --- | --- | --- | --- | --- | --- | --- | --- |

| 1330 | gi\|71895153 | NADH dehydrogenase (ubiquinone) 1 α subcomplex, 10, 42kDa | NDUFA10 | 41633 | 6.15 | 27% | 90/72 | 8/19 | YFPEADIHYQDR,LFVQDKDGVLDPVAIPR | −1.11 | 1 | 1.59 | Mitochondrion | Energy production |
| --- | --- | --- | --- | --- | --- | --- | --- | --- | --- | --- | --- | --- | --- | --- |

| 1790 | gi\|71895681 | Electron transfer flavoprotein, α polypeptide | ETFA | 36999 | 8.87 | 29% | 86/72 | 7/19 | LLYDLADQLNAAVGASR,RLGGEVSCLVAGTSCDK | −1.05 | 1.2 | 2.12 | Mitochondrion | Energy production |
| --- | --- | --- | --- | --- | --- | --- | --- | --- | --- | --- | --- | --- | --- | --- |

| 898 | gi\|71895387 | Inosine monophosphate dehydrogenase 2 | IMPDH2 | 55923 | 6.57 | 31% | 138/72 | 14/38 | QLLCGAAIGTHEDDKYR,TTSAQVEGGVHGLHSYEK | −1.02 | −1.04 | 1.63 | No annotation | DNA synthesis |
| --- | --- | --- | --- | --- | --- | --- | --- | --- | --- | --- | --- | --- | --- | --- |
| 1887 | gi\|88909244 | Prohibitin | PHB | 29935 | 5.57 | 65% | 146/69 | 14/46 | FGLGLAVAGGVVNSALYNVDAGHR,AAELIANSLATAGDGLIELR | −1 | 1.13 | 2.45 | Mitochondrion | DNA synthesis |

| 1103 | gi\|46048767 | α-Enolase | ENO1 | 47617 | 6.17 | 29% | 98/69 | 11/33 | AAVPSGASTGIYEALELR,YNQLLR | 1.3 | −1.09 | -2.91 | Cytosol | Metabolism (carbohydrate) |
| --- | --- | --- | --- | --- | --- | --- | --- | --- | --- | --- | --- | --- | --- | --- |

| 1443 | gi\|1703245 | Fructose-bisphosphate aldolase C (fragment) | ALDOC | 14543 | 6.13 | 52% | 67/64 | 6/44 | YSPEEIAMATVTALR,DNANAATEEFVKR | 1.18 | −1.03 | −2.25 | Cytoplasm | Metabolism (carbohydrate) |
| --- | --- | --- | --- | --- | --- | --- | --- | --- | --- | --- | --- | --- | --- | --- |

| 1460 | gi\|1703246 | Fructose-bisphosphate aldolase C (fragment) | ALDOC | 14543 | 6.13 | 52% | 74/61 | 7/58 | DNANAATEEFVKR | 1.14 | −1.01 | −3.19 | Cytoplasm | Metabolism (carbohydrate) |
| --- | --- | --- | --- | --- | --- | --- | --- | --- | --- | --- | --- | --- | --- | --- |

| 1565 | gi\|2506442 | Glyceraldehyde-3-phosphate dehydrogenase | GAPDH | 35909 | 8.71 | 23% | 75/69 | 8/32 | LVINGHAITIFQERDPSNIK,VPTPNVSVVDLTCR | 1.08 | 1.18 | 1.68 | Cytoplasm/nucleus | Metabolism (carbohydrate) |
| --- | --- | --- | --- | --- | --- | --- | --- | --- | --- | --- | --- | --- | --- | --- |

| 1907 | gi\|82082619 | Phosphoglycerate mutase 1 | PGAM1 | 29051 | 7.03 | 44% | 88/69 | 8/30 | VLIAAHGNSLR,FCGWYDADLSPAGQQEAR | 1.35 | 1.02 | −3.12 | Cytosol | Metabolism (carbohydrate) |
| --- | --- | --- | --- | --- | --- | --- | --- | --- | --- | --- | --- | --- | --- | --- |
| 1939 | gi\|136056 | Triosephosphate isomerase | TPI1 | 26832 | 6.71 | 66% | 203/69 | 15/50 | HVFGESDELIGQK,TATPQQAQEVHEK | 1.21 | −1.04 | −2.69 | Cytosol | Metabolism (carbohydrate) |

| 763 | gi\|57524955 | Acyl-coenzyme A dehydrogenase family, member 9 | ACAD9 | 68049 | 7.94 | 17% | 87/72 | 10/21 | TAAPRPAYAK,VAMNILNSGR | 1.07 | 1.17 | 1.87 | Mitochondrion | Metabolism (lipid) |
| --- | --- | --- | --- | --- | --- | --- | --- | --- | --- | --- | --- | --- | --- | --- |

| 928 | gi\|60592998 | Succinyl-CoA: 3-ketoacid-coenzyme A transferase 1 | OXCT1 | 56549 | 8.01 | 36% | 91/72 | 14/49 | GITAVSNNAGVDNFGLGLLLQTK,GLTLVEIWEGLSVDDIK | −1.16 | −1.03 | 1.85 | Mitochondrion | Metabolism (lipid) |
| --- | --- | --- | --- | --- | --- | --- | --- | --- | --- | --- | --- | --- | --- | --- |

| 1248 | gi\|57529797 | Acyl-coenzyme A dehydrogenase, long chain | ACADL | 48259 | 8.34 | 31% | 86/72 | 14/48 | KIFSSDHDIFR,CIGAIAMTEPGAGSDLQGVR | 1.01 | 1.08 | 1.58 | Mitochondrion | Metabolism (lipid) |
| --- | --- | --- | --- | --- | --- | --- | --- | --- | --- | --- | --- | --- | --- | --- |

| 1349 | gi\|3915686 | Farnesyl pyrophosphate synthetase | FDPS | 42583 | 6.16 | 25% | 107/69 | 9/20 | EGVGLDAINDSFLLESSVYR,QLLEDNYGRK | 1.26 | −1.02 | −1.74 | Cytoplasm | Metabolism (lipid) |
| --- | --- | --- | --- | --- | --- | --- | --- | --- | --- | --- | --- | --- | --- | --- |

| 1258 | gi\|57529515 | Ornithine aminotransferase | OAT | 48825 | 7.12 | 32% | 113/72 | 12/29 | TIQGPPSSDYIFER,MLAVDHENVRPDIILLGK | 1.03 | 1.01 | 1.52 | No annotation | Metabolism (protein) |
| --- | --- | --- | --- | --- | --- | --- | --- | --- | --- | --- | --- | --- | --- | --- |

| 1827 | gi\|67476967 | Endoplasmic reticulum protein ERp29 (Fragment) | ERP29 | 25471 | 7.66 | 28% | 68/61 | 6/33 | FMSVTDKSER | −1.08 | 1.04 | 1.56 | Endoplasmic reticulum | Chaperone |
| --- | --- | --- | --- | --- | --- | --- | --- | --- | --- | --- | --- | --- | --- | --- |

| 1087 | gi\|61217546 | Spliceosome RNA helicase BAT1 | DDX39B | 49428 | 5.44 | 23% | 71/61 | 9/34 | DFLLKPELLR,LTLHGLQQYYVK | 1.12 | −1.04 | −1.76 | Nucleus | RNA processing |
| --- | --- | --- | --- | --- | --- | --- | --- | --- | --- | --- | --- | --- | --- | --- |

| 1415 | gi\|166223489 | Serine-threonine kinase receptor-associated protein | STRAP | 38402 | 4.98 | 36% | 96/69 | 9/27 | QGDTGDWIGTFLGHK,TIAFHSAETLEQIK | 1.06 | −1.06 | −2.01 | Cytoplasm/nucleus | RNA processing |
| --- | --- | --- | --- | --- | --- | --- | --- | --- | --- | --- | --- | --- | --- | --- |

| 970 | gi\|57525441 | FK506 binding protein 4, 59kDa | FKBP4 | 50742 | 5.52 | 23% | 75/72 | 10/37 | KGEGYLKPNEGALVEIQFEGR,GEGYLKPNEGALVEIQFEGR | 1.11 | −1.08 | −2.36 | Cytoplasm/nucleus | Signal transduction |
| --- | --- | --- | --- | --- | --- | --- | --- | --- | --- | --- | --- | --- | --- | --- |

| 1088 | gi\|45384364 | GDP dissociation inhibitor 2 | GDI2 | 51107 | 5.23 | 40% | 137/81 | 15/26 | VICILSHPIK,MLLYTEVTR | 1.12 | −1.04 | −1.76 | Golgi | Signal transduction |
| --- | --- | --- | --- | --- | --- | --- | --- | --- | --- | --- | --- | --- | --- | --- |

| 1739 | gi\|1351941 | Annexin A5 | ANXA5 | 36290 | 5.6 | 56% | 164/69 | 18/46 | FETLMVSLMRPAR,WGTDEETFITILGTR | 1.05 | −1.08 | −1.94 | No annotation | Signal transduction |
| --- | --- | --- | --- | --- | --- | --- | --- | --- | --- | --- | --- | --- | --- | --- |

| 1766 | gi\|54037169 | Guanine nucleotide-binding protein subunit β-2-like 1 | GNB2L1 | 35511 | 7.6 | 39% | 121/69 | 10/24 | DVLSVAFSSDNR,YTVQDESHSEWVSCVR | 1.02 | −1.02 | −1.64 | Cytoplasm/nucleus | Signal transduction |
| --- | --- | --- | --- | --- | --- | --- | --- | --- | --- | --- | --- | --- | --- | --- |

| 1860 | gi\|82197872 | 14-3-3 protein β/α | YWHAB | 28004 | 4.76 | 29% | 61/62 | 10/50 | QTTVANSQQAYQEAFEISK,MKGDYYR | 1.1 | −1.03 | −2.19 | Cytoplasm | Signal transduction |
| --- | --- | --- | --- | --- | --- | --- | --- | --- | --- | --- | --- | --- | --- | --- |

| 1921 | gi\|2506057 | Calpain small subunit | unnamed | 24014 | 5.05 | 37% | 84/72 | 6/18 | YLWNNIK,YGDEGGNLDFDNFISCLVR | −1.02 | −1.1 | −1.56 | Cytoplasm | Signal transduction |
| --- | --- | --- | --- | --- | --- | --- | --- | --- | --- | --- | --- | --- | --- | --- |

| 1965 | gi\|82080783 | Small ubiquitin-related modifier 3 | SUMO3 | 10818 | 5.34 | 26% | 62/61 | 4/11 | VAGQDGSVVQFK,VAGQDGSVVQFKIK | 1.1 | −1.05 | −1.64 | Cytoplasm | Signal transduction |
| --- | --- | --- | --- | --- | --- | --- | --- | --- | --- | --- | --- | --- | --- | --- |

| 348 | gi\|123668 | Heat shock protein HSP 90-α | HSP90AA1 | 84406 | 5.01 | 21% | 89/69 | 15/34 | SLTNDWEDHLAVK,DNSTMGYMAAKK | 1.22 | −1.12 | −2.23 | Cytoplasm | Stress response |
| --- | --- | --- | --- | --- | --- | --- | --- | --- | --- | --- | --- | --- | --- | --- |
| 604 | gi\|82190481 | Heat shock cognate 71 kDa protein | HSPA8 | 71011 | 5.47 | 33% | 137/69 | 14/38 | VEIIANDQGNR,ITITNDKGR | 1.1 | −1.11 | −1.67 | Cytoplasm | Stress response |

| 639 | gi\|57525126 | TNF receptor-associated protein 1 | TRAP1 | 80082 | 6.64 | 30% | 121/81 | 19/40 | SIFYVPEQKPSMFDISR,YESSALPAGQLTSLTEYASR | −1.02 | 2.54 | 3.3 | Cytoplasm/nucleus | Signal transduction |
| --- | --- | --- | --- | --- | --- | --- | --- | --- | --- | --- | --- | --- | --- | --- |

| 812 | gi\|82197843 | 60 kDa heat shock protein, mitochondrial | HSPD1 | 61105 | 5.72 | 28% | 111/69 | 15/42 | TVIIEQSWGSPK,GIIDPTKVVR | 1.01 | 1.15 | 2.46 | Mitochondrion | Stress response |
| --- | --- | --- | --- | --- | --- | --- | --- | --- | --- | --- | --- | --- | --- | --- |

| 1453 | gi\|187609623 | Eukaryotic translation initiation factor 2 subunit 1 | EIF2S1 | 36441 | 5.07 | 22% | 63/61 | 7/19 | RPGYGAYDAFK,INLIAPPR | 1.19 | 1.03 | −2.02 | Cytoplasm/nucleus | Translation |
| --- | --- | --- | --- | --- | --- | --- | --- | --- | --- | --- | --- | --- | --- | --- |

| 1838 | gi\|1710781 | 40S ribosomal protein SA | RPSA | 33115 | 4.8 | 18% | 62/61 | 4/16 | FTPGTFTNQIQAAFR,AIVAIENPADVSVISSR | −1.41 | 1 | 9.54 | Cytoplasm/nucleus | Translation |
| --- | --- | --- | --- | --- | --- | --- | --- | --- | --- | --- | --- | --- | --- | --- |

| 2300 | gi\|93004707 | Eukaryotic translation initiation factor 5A-1 | EIF5A1 | 16164 | 5.32 | 50% | 63/62 | 4/32 | VHLVGIDIFTGK,CDFQLIGIQDGFLSLLQDSGEVR | 1.34 | −1 | −2.66 | Cytoplasm/nucleus | Translation |
| --- | --- | --- | --- | --- | --- | --- | --- | --- | --- | --- | --- | --- | --- | --- |

| 1127 | gi\|88908538 | 26S proteasome non-ATPase regulatory subunit 13 | PSMD13 | 43009 | 5.37 | 24% | 64/61 | 9/44 | VHMTWVQPR,DRLEFWCTDVR | 1.06 | −1.23 | −2.29 | Proteasome | Ubiquitin-proteasome Pathway |
| --- | --- | --- | --- | --- | --- | --- | --- | --- | --- | --- | --- | --- | --- | --- |

| 1780 | gi\|82075143 | Proteasome activator complex subunit 3 | PSME3 | 29520 | 5.85 | 40% | 103/69 | 9/26 | IEDGNNFGVSIQEETVAELR | 1.1 | −1.23 | −3.55 | Proteasome | Ubiquitin-proteasome Pathway |
| --- | --- | --- | --- | --- | --- | --- | --- | --- | --- | --- | --- | --- | --- | --- |

| 1802 | gi\|57529900 | Proteasome (prosome, macropain) subunit, α type, 3 | PSMA3 | 28691 | 4.93 | 29% | 72/72 | 7/18 | HVGMAVAGLLADAR,LYEEGSNKR | 1.19 | 1.06 | −2.3 | Proteasome | Ubiquitin-proteasome Pathway |
| --- | --- | --- | --- | --- | --- | --- | --- | --- | --- | --- | --- | --- | --- | --- |

| 607 | gi\|54399721 | μB [Avian orthoreovirus] | μB | 73568 | 5.31 | 30% | 192/69 | 17/36 | LQMACELDRDYLDAR,LVNDVGVDIVCSR | −1.1 | 2.88 | 5.96 | Viral protein | Viral protein |
| --- | --- | --- | --- | --- | --- | --- | --- | --- | --- | --- | --- | --- | --- | --- |

| 615 | gi\|185812894 | μNS protein [Avian orthoreovirus] | μNS | 71360 | 5.88 | 35% | 172/69 | 19/43 | QSGGQLLEHYR,WAIDHACTDSLVSTR | 2.6 | 16.17 | 12.16 | Viral protein | Viral protein |
| --- | --- | --- | --- | --- | --- | --- | --- | --- | --- | --- | --- | --- | --- | --- |

| 629 | gi\|54399719 | μB [Avian orthoreovirus] | μB | 73610 | 5.55 | 28% | 177/69 | 16/20 | AWVLIDSSLNASDPSSLR,IIGETSLSYLFER | 1.13 | 4.41 | 7.35 | Viral protein | Viral protein |
| --- | --- | --- | --- | --- | --- | --- | --- | --- | --- | --- | --- | --- | --- | --- |
| 636 | gi\|45862453 | μNS protein [Avian orthoreovirus] | μNS | 71374 | 5.88 | 42% | 198/69 | 24/45 | LDEEEYNVIR,ADSTGWCVYR | 2.66 | 12.26 | 8.39 | Viral protein | Viral protein |

| 638 | gi\|185812859 | μB [Avian orthoreovirus] | μB | 73659 | 5.31 | 39% | 220/69 | 23/46 | GVSPESVNIHNYIVYVDCFVGVSAR,VEEALWLR | 1.62 | 14.67 | 29.73 | Viral protein | Viral protein |
| --- | --- | --- | --- | --- | --- | --- | --- | --- | --- | --- | --- | --- | --- | --- |

| 895 | gi\|45862453 | μNS protein [Avian orthoreovirus] | μNS | 71374 | 5.88 | 33% | 172/69 | 18/32 | VCVGIDPLVTR,DELLQEVDELKR | −1.09 | 1.22 | 15.83 | Viral protein | Viral protein |
| --- | --- | --- | --- | --- | --- | --- | --- | --- | --- | --- | --- | --- | --- | --- |

| 1351 | gi\|12006927 | σNS [Avian orthoreovirus] | σNS | 40794 | 6.72 | 27% | 94/69 | 9/30 | IMLAMMAAQLK,HNNVKQILTR | 1.11 | 2.09 | 2.03 | Viral protein | Viral protein |
| --- | --- | --- | --- | --- | --- | --- | --- | --- | --- | --- | --- | --- | --- | --- |

| 1369 | gi\|9651567 | σB protein [Avian orthoreovirus] | σB | 41595 | 6.27 | 42% | 169/69 | 17/49 | DIAASCWR | 3.44 | 27.12 | 61.44 | Viral protein | Viral protein |
| --- | --- | --- | --- | --- | --- | --- | --- | --- | --- | --- | --- | --- | --- | --- |

| 1375 | gi\|9651565 | σB protein [Avian orthoreovirus] | σB | 41671 | 6.16 | 53% | 147/69 | 18/49 | TPACWNAQTAWDTVTFHVPDVIR,DIAASCWR | 2.09 | 17.62 | 43.43 | Viral protein | Viral protein |
| --- | --- | --- | --- | --- | --- | --- | --- | --- | --- | --- | --- | --- | --- | --- |

| 1448 | gi\|55583977 | σC capsid protein | σC | 34948 | 5 | 27% | 66/53 | 8/37 | AGLNPSQR,VFTITFPTGGDGTANIR | 1.33 | 3.67 | 11.55 | Viral protein | Viral protein |
| --- | --- | --- | --- | --- | --- | --- | --- | --- | --- | --- | --- | --- | --- | --- |

^a^Number given to unique protein spots in the 2D gel.

^b^NCBI genInfo identifier for sequences matching MALDI-TOF/MS-identified proteins searched with MASCOT against the non-redundant protein database.

^c^Number of amino acids spanned by the assigned peptides divided by the sequence length.

^d^Average ratio of differential expression (*p* < 0.05) between 24 , 48, or 72 h post-infection and mock-infected cells (Mock) calculated from triplicate gels.

^e^Subcellular locations and functional classes of identified proteins obtained from Uniprot (<http://www.uniprot.org/>).

MW: molecular weight; p*I*: isoelectric point.
